# Environmental Factors Shape Water Microbial Community Structure and Function in Shrimp Cultural Enclosure Ecosystems

**DOI:** 10.3389/fmicb.2017.02359

**Published:** 2017-11-29

**Authors:** Dongwei Hou, Zhijian Huang, Shenzheng Zeng, Jian Liu, Dongdong Wei, Xisha Deng, Shaoping Weng, Zhili He, Jianguo He

**Affiliations:** ^1^State Key Laboratory of Biocontrol, Guangdong Provincial Key Laboratory of Marine Resources and Coastal Engineering, School of Marine Sciences, Sun Yat-sen University, Guangzhou, China; ^2^School of Life Sciences, Sun Yat-sen University, Guangzhou, China; ^3^School of Environmental Science and Engineering, Sun Yat-sen University, Guangzhou, China; ^4^Department of Botany and Microbiology, Institute for Environmental Genomics, University of Oklahoma, Norman, OK, United States

**Keywords:** aquatic microbial community, microbial community function, shrimp cultural enclosure ecosystems, salinity, environmental factors

## Abstract

The effects of environmental factors on water microbial communities have been extensively studied, but little is known about the effects in shrimp cultural enclosure ecosystems. We analyzed 16S rRNA gene amplicons to determine the principal environmental factors that shape the structure and function of microbial communities in shrimp cultural enclosure ecosystems from Guangdong and Hainan provinces, in China. High quality sequences were clustered into operational taxonomic units (OTUs) at the 97% similarity level, generating 659–1,835 OTUs per sample. The 10 most abundant phyla were Proteobacteria, Bacteroidetes, Cyanobacteria, Planctomycetes, Actinobacteria, Verrucomicrobia, Firmicutes, Chlorobi, Chloroflexi, and Chlamydiae. The results of canonical correspondence analyses (CCA) indicated that salinity, total phosphate (TP), total nitrogen (TN), temperature, and pH were the most important factors shaping microbial community structure. Differences in microbial community structure between high and low salinity samples were explained by changes in the relative abundances of some OTUs (e.g., OTU5, OTU19, OTU21, OTU39, and OTU71). Moreover, the contribution of spatial distribution to the microbial community assembly was investigated via aggregated boosted tree (ABT) analyses, and the results indicated spatial isolation was not a major factor affecting the phylogenetic diversity and phylotypes of water microbial communities. Furthermore, we predicted water microbial community functional profiling using the PICRUSt program and principal component analyses (PCA) suggested that salinity was a major contributor to the structure and function of the microbial communities. Collectively, these results showed that environmental factors influenced the structure and function of water microbial communities, while salinity was the principal environmental factor instead of temperature, TP, TN, and pH in shrimp cultural enclosure ecosystems.

## Introduction

Environmental factors can affect microbial communities both in terms of structure (Allison and Martiny, 2008) and function (Shade et al., 2012a,b). Understanding which environmental factors influence the microbial community is a key goal of microbial ecology (Green et al., 2008). Many studies have shown that environmental factors, such as salinity (Székely et al., 2013), temperature (Sunagawa et al., 2015), organic matter (Dang and Lovell, 2016), and pH (Liu et al., 2015), influence microbial community structure in different ecosystems.

A growing number of studies have addressed the patterns and functions of microbial communities in the waters of different ecosystems. A previous study determined that after a salinity perturbation, the microbial response depended on the recruitment of specific taxa, including *Loktanella, Erythrobacter*, and *Rheinheimera*, and these changes were associated with alterations of the microbial community structure in response to the main functional contributor, salinity (Comte et al., 2017). In the waters of coastal lakes, the distribution of both abundant and rare microbial taxa was also influenced by the range of salinity (Logares et al., 2013). Environmental conditions (e.g., temperature) strongly influence the microbial community structure and distribution of functional groups by shaping metabolic niches in marine ecosystems (Louca et al., 2016). Moreover, organic matter (in both particulate and dissolved forms) may be a major factor that controls the composition and structure of microbial communities in marine waters (Dang and Lovell, 2016). Another study suggested that local environmental factors (e.g., temperature, and pH) influenced microbial community patterns in Crucian Carp (*Carassius auratus*) culture ponds (Li et al., 2017). Therefore, these studies provided new insights into the patterns of microbial communities in water, and they improved our understanding of how microbial communities respond to environmental changes.

Enclosed aquaculture is an important fishery production mode that, has been the most important driver for total increases in annual aquaculture output, and this subsector contributed to a 65% increase global farmed food aquatic production (FAO, 2015). Because this aquaculture mode results in local environmental eutrophication, enclosed aquaculture ponds face severe problems, particularly microbial disease outbreaks (Chifumi et al., 2005; Choi et al., 2016) and associated impacts on surrounding environments (Sakami et al., 2008). In order to solve these problems, an increasing number of studies have focused on the microbial communities in aquaculture environments (Santander-de Leon et al., 2015; Dabade et al., 2016; Fan et al., 2016). However, few reports have explored the correlations between the structure and function of microbial communities and the environmental factors in shrimp cultural enclosure ecosystems.

In this study, we identified the principal environmental factors that shape the structure and function of microbial communities in shrimp cultural enclosure ecosystems. Previous analyses demonstrated that salinity (Lozupone and Knight, 2007; Auguet et al., 2010) and temperature (Székely et al., 2013; Sunagawa et al., 2015; Comte et al., 2017) are key environmental factors that globally shape the structure and function of microbial communities in different environments. Therefore, we hypothesized that both salinity and temperature play a major role in shaping the microbial community structure and function in shrimp enclosure ecosystems. To test these hypotheses, we examined water microbial communities in an enclosed aquaculture ecosystem, and we explored whether predictable relationships occurred between microbial communities and specific environmental factors [e.g., salinity, temperature, pH, total phosphate (TP), and total nitrogen (TN)]. High-throughput sequencing of 16S rRNA gene amplicons and a series of statistical analysis methods were employed to generate reliable and sufficient information about the water microbial communities in shrimp enclosed aquaculture ecosystem.

## Materials and methods

### Sample collection and physicochemical analysis

**Figure 1:**
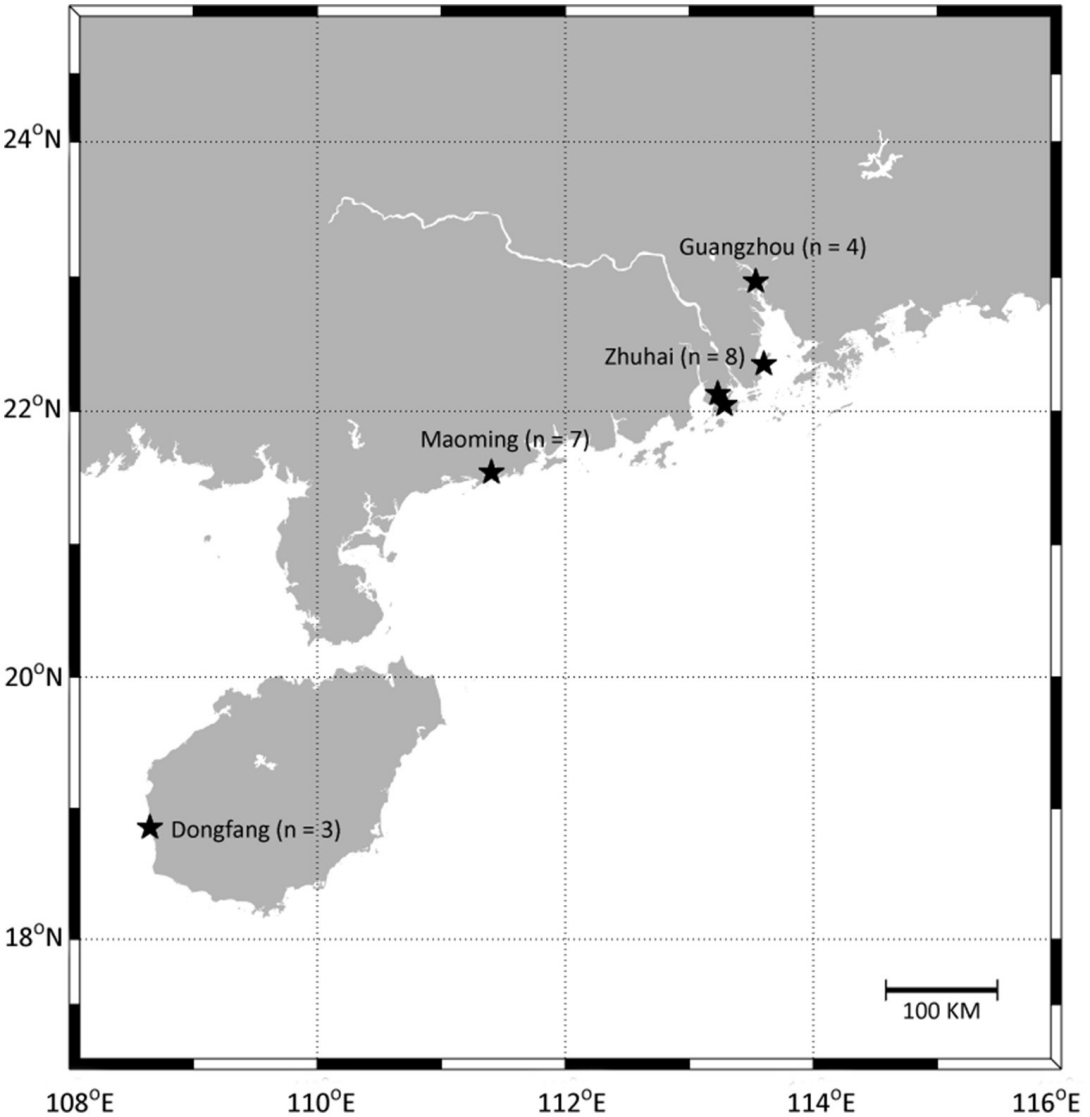
Location of sampling sites. Samples were collected in Maoming City, Zhuhai City, Guangzhou City, and Dongfang City, respectively. Detailed sites characteristics are listed in Table S1.

A total of 66 water samples (from 22 enclosed culture ponds with three replicate) were collected from four pacific white shrimp cultural regions in Guangdong and Hainan provinces, China (Figure 1, Table S1). The sampled ponds were of similar size, water depth, and shrimp stocking density. Samples A, B, C, D, E, F, G, T, U, and V were from high salinity cultural areas in Maoming and Dongfang City, and samples H, I, J, K, L, M, N, O, P, Q, R, and S were from low salinity cultural areas in Zhuhai and Guangzhou City. Site locations were recorded via the global positioning system (GPS, Garmin Vista HCx) and the geographical distances between sampling sites ranged from about 50 m to over 683 km. For each sample, 1.0 L of water was taken from a depth of 0.5 m below the surface using sterile bottles, and samples were immediately placed on ice (Hou et al., 2016) before being filtered through a 0.22 μm polyethersulfone membrane filter (Supor-200, Pall) using a peristaltic pump. The cell pellets on the polyethersulfone membranes were stored at −80°C until DNA extraction. Temperature, pH, DO, and salinity were measured on-site using a YSI handheld multi-parameter instrument (Model YSI 380, YSI Incorporated, USA). For the chemical analyses, 200 mL of each sample was collected from the same location using sterile bottles. The samples were placed on ice, immediately transported to the laboratory, and stored at 4°C. TN, TP concentrations of dissolved inorganic nitrogen (${\text{NH}}_{4}^{+}$-N, ${\text{NO}}_{2}^{-}$-N, and ${\text{NO}}_{3}^{-}$-N), and orthophosphate (${\text{PO}}_{4}^{3-}$-P) were measured using an automatic discrete analyzer (Model CleverChem 200, DeChem-Tech, Germany).

### DNA extraction, amplification, and sequencing

DNA was extracted from all samples using a Water DNA Kit (Omega Bio-tek, USA) base on the manufacturer's instructions. The concentration and purity of genomic DNA was determined using a NanoVuePlus Spectrophotometer (GE Healthcare, USA). Regarding the 16S rRNA gene amplicon analysis, the V4 hypervariable region of the 16S rRNA gene was amplified using 515F/806R primers (Bates et al., 2010). PCR reactions were carried out using Phusion High-Fidelity PCR Master Mix (New England Biolabs, UK), and PCR products were pooled at equimolar concentrations. Sequencing libraries were generated using a TruSeq DNA PCR-Free Sample Preparation Kit (Illumina, USA), and index codes were added. The libraries were sequenced with the Illumina HiSeq2500 system (Illumina, USA) at MAGIGENE Biological Technology Co. Ltd (Guangzhou, China).

### Processing of sequencing data

Raw data generated from the HiSeq2500 system were merged using FLASH (Version 1.2.7) (Magoc and Salzberg, 2011). In order to control the quality process, raw tags were then filtered to obtain high-quality clean tags using the Quantitative Insights into Microbial Ecology (QIIME) software package (Version 1.7.0) (Bates et al., 2010; Bokulich et al., 2013). To detect and remove chimeric sequences, the tags were compared to the Gold database (http://drive5.com/uchime/uchime_download.html) using the UCHIME algorithm (http://www.drive5.com/usearch/manual/uchime_algo.html) (Edgar et al., 2011), resulting in a set of effective tags. OTU (97% similarity) sequences were aligned using Uparse (Version 7.0.1001), and the Greengenes Database (DeSantis et al., 2006) was used to annotate taxonomic information for each representative sequence using the RDP classifier (Wang et al., 2007). To determine the phylogenetic relationships of different OTUs and the dominant species in different samples, multiple sequence alignments were conducted using MUSCLE (Version 3.8.31) (Edgar, 2004). OTU abundance information was normalized with a standard sequence number that corresponded to a sample that contained the least number of sequences (Cai et al., 2014). Chao1 (richness estimate) and Shannon (diversity index) estimates were calculated based on the observed species, and analyses were conducted in QIIME (Version 1.7.0).

### Statistical analysis

All statistical analyses were implemented using various packages within the R statistical computing environment. The unweighted pair-group method with arithmetic mean analysis (UPGMA) was performed to reveal the similarity of the 66 samples using an open source software package. An aggregated boosted tree (ABT) analysis was carried out using the *gbmplus* package to quantitatively evaluate the relative influence of environmental variables on microbial community diversity (De'ath, 2007). Multiple linear regression (MLR) with the stepwise method and a Mantel test were conducted to determine the significance between diversity indexes and site properties, and analyses were conducted using vegan package (version 1.17-0). Regarding the phylogenetic diversity dataset (the sum of all branches in the phylogenetic tree of the community and species number only) and phylotype (species number, regardless of species abundance) in the subsequent MLR analyses, independent variables (including salinity, temperature, pH, dissolved oxygen (DO), TN, TP, ${\text{NH}}_{4}^{+}$-N, ${\text{NO}}_{2}^{-}$-N, ${\text{NO}}_{3}^{-}$-N, and ${\text{PO}}_{4}^{3-}$-P), latitude, and longitude were input into the MLR model. To examine the relationship between microbial community structure and environmental factors, canonical correspondence analyses (CCA) were conducted using CANOCO software (Dang et al., 2010). An ANOVA followed by stepwise ordination was used to determine the significance of the overall model used to create the CCA and to obtain the significance of *P-*values for each of the environmental variables included. The most differentially abundant taxa (at both OTU and genera levels) between the high and low salinity samples were identified using Welch's *t-*test. The functional microbiota content was predicted from the 16S rRNA gene sequence data using PICRUSt software (Langille et al., 2013). A closed reference OTU picking strategy was used, removing all of the OTUs that did not match the GreenGenes 13_−_5 reference sequences at 97% similarity. OTUs were normalized by dividing their abundances by known or predicted 16S rRNA gene copy number abundances before final metagenomic predictions were made. Predicted functional counts were rarefied to the same depth, and single ANOVA analyses were calculated by comparing Kyoto Encyclopedia of Genes and Genomes (KEGG) orthologs values (levels 1, 2, and 3) in both low and high salinity samples. A principal component analysis (PCA) was also conducted to analyze the similarity of microbial community function content based on metagenomic functional prediction consequences (level 3). Moreover, the most differentially abundant functional genes between the high and low salinity samples were identified using Welch's *t*-test.

## Results

### Environmental factors

The water samples from the 22 ponds captured a wide range of physical and geochemical gradients (Table S2), and samples were characterized based on salinity (0.42–32.71‰) and DO (3.45–19.04 mg^.^L^−1^). Water temperatures ranged from 27.70 to 32.40°C, and pH-values ranged from 7.31 to 9.81. ${\text{NH}}_{4}^{+}$-N, ${\text{NO}}_{2}^{-}$-N, ${\text{NO}}_{3}^{-}$-N and ${\text{PO}}_{4}^{3-}$-P were in ranges of 0.01–3.24, 0.01–0.37, 0.01–2.08, and 0.02–0.58 mg^.^L^−1^ respectively. Additionally, TN (0.35–5.16 mg^.^L^−1^) and TP (0.01–1.32 mg^.^L^−1^) fluctuated considerably.

### Microbial community diversity and structure

HiSeq sequencing of 16 rRNA gene amplicons generated 3,367,470 quality sequences from a total of 66 samples, and there was an average of 51,022 sequences (43,116–59,029) per sample (Table S3). A total of 2,450,746 gene fragments were selected for classification, and fragments were subsequently clustered into 9,988 prokaryotic operational taxonomic units (OTUs). The numbers of OTUs detected in each sample ranged from 659 to 1,835. The following diversity indices were calculated based on the OTUs of each library: Good's coverage index ranged from 0.95 to 0.97; the Shannon index ranged from 3.48 to 7.94; and Chao1 was from 702 to 2,593 (higher than the actual number of observed OTUs in each library).

**Figure 2:**
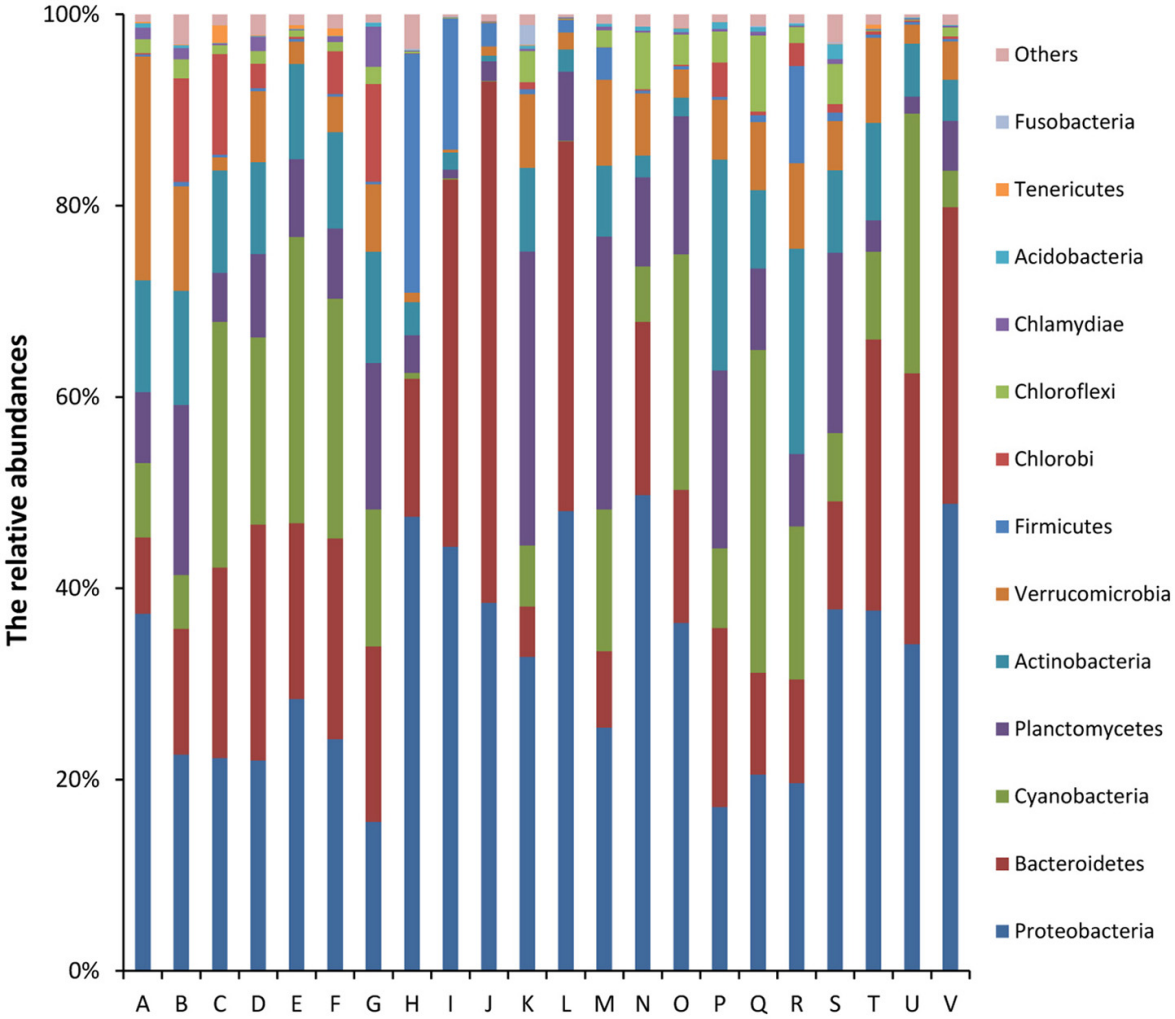
Relative abundances (%) of dominant phyla from all samples based on 16S rRNA gene amplicon sequencing data. The phylum level distribution presented is based on 80% similarity cluster OTUs. Unclassified phyla with relative abundances lower than 1% were assigned as “others.” The 10 major dominant phyla included Proteobacteria, Bacteroidetes, Cyanobacteria, Planctomycetes, Actinobacteria, Verrucomicrobia, Firmicutes, Chlorobi, Chloroflexi, and Chlamydiae.

Of the classifiable sequences, 58 phyla were identified. The most dominant community members, Proteobacteria, Bacteroidetes, Cyanobacteria, Planctomycetes, Actinobacteria, Verrucomicrobia, Firmicutes, Chlorobi, Chloroflexi, and Chlamydiae accounted for 32.12, 20.58, 12.61, 10.61, 8.35, 5.99, 2.81, 2.44, 1.94, and 0.73% of the total microbial community, respectively (Figure 2). Acidobacteria (0.33%), Tenericutes (0.15%), Fusobacteria (0.13%), Gemmatimonadetes (0.10%), and Spirochaetes (0.03%) were less abundant, but the phyla were detected in all samples. At the genus level, the most abundant phylotypes were affiliated with Synechococcus, *Flavobacterium, Pseudomonas, Paenisporosarcina, Rheinheimera, Janthinobacterium, Luteolibacter, Shewanella, Mycoplana*, and *Rhodobacter*, which accounted for 8.76, 6.05, 1.73, 1.40, 1.25, 1.11, 1.08, 0.98, 0.97, and 0.93% of the total microbial community, respectively (Figure S1).

### Similarity analysis and effects of environmental factors on microbial community

**Figure 3:**
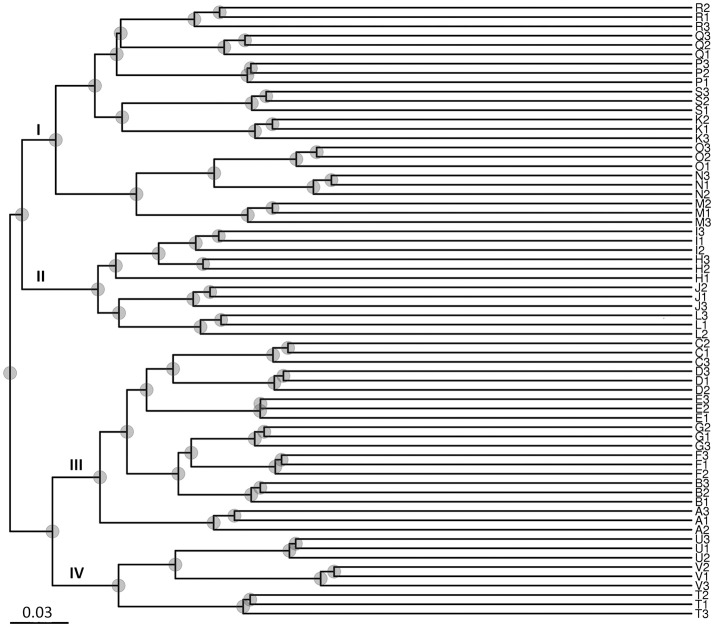
Unweighted Pair-Group Method with Arithmetic Means (UPGMA) analysis of microbial community structure based on 16S rRNA gene amplicon sequencing data. Group I contained eight sites (K–O from Zhuhai City; and P–S from Guangzhou City); Group II contained four sites (H–K from Zhuhai City); Group III contained seven sites (A–G from Maoming City); Group IV contained three sites (T–V from Dongfang City). Groups I and II were associated with low salinity samples, and groups III and IV were associated with high salinity samples.

The results of the UPGMA analysis indicated that the samples were clustered into four groups (Figure 3). Group I contained eight sites (K, M, N, O, P, Q, R, and S), group II contained four sites (H, I, J, and K), group III contained seven sites (A, B, C, D, E, F, and G), and group IV contained three sites (T, U, and V). Groups I and II corresponded to low salinity samples, and groups III and IV corresponded to high salinity samples, which were largely associated with salinity instead of locations/sites. Therefore, the results suggested that salinity might be a major factor shaping water microbial community divergence in such enclosed aquaculture ecosystems.

**Figure 4:**
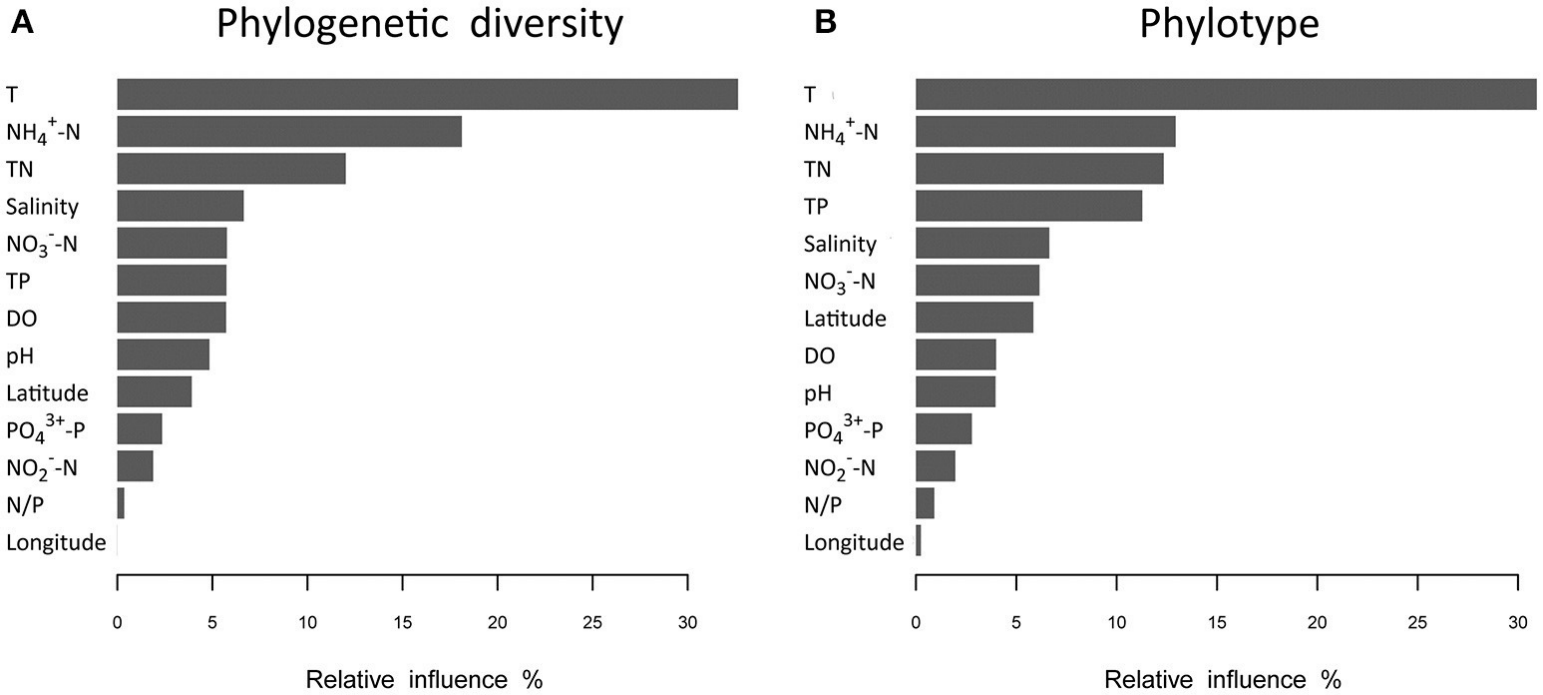
Relative influence (%) of environmental properties and spatial distance (represented by latitudinal and longitudinal) on phylogenetic diversity **(A)** and phylotypes **(B)** that were evaluated using aggregated boosted tree (ABT) models. Temperature was a major factor associated with patterns of both phylogenetic diversity and phylotypes in aquaculture ponds, and it accounted for over 30% of the relative influence. The geographical distance (represented latitudinal and longitudinal) had no obvious effects on the phylogenetic diversity and phylotypes of water microbial communities.

**Figure 5:**
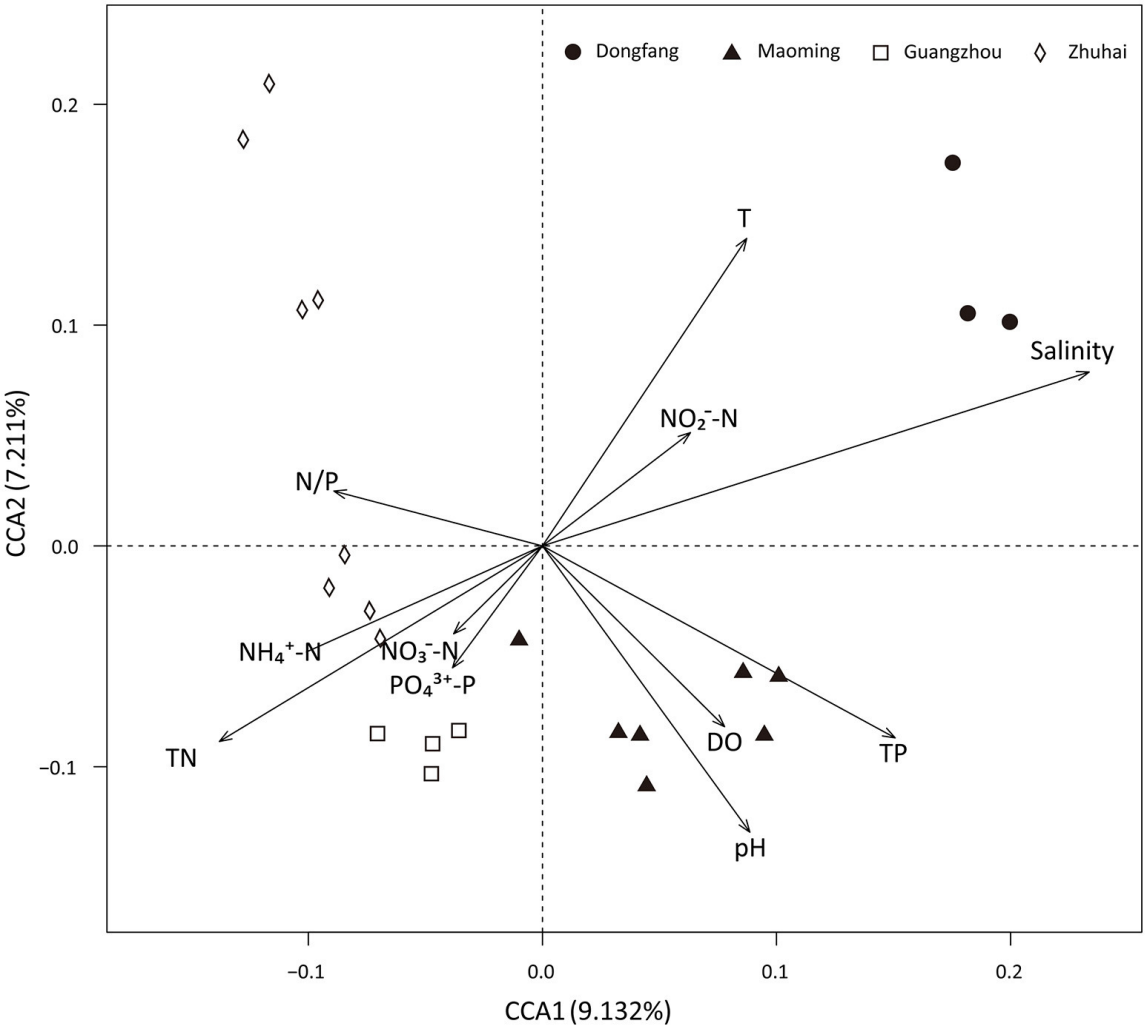
Canonical correspondence analysis (CCA) of the microbial community structure based on 16S rRNA gene amplicon sequencing data. Salinity (*r*^2^ = 0.971, *P* = 0.001), TP (*r*^2^ = 0.473, *P* = 0.004), temperature (*r*^2^ = 0.423, *P* = 0.007), TN (*r*^2^ = 0.428, *P* = 0.005), and pH (*r*^2^ = 0.380, *P* = 0.016) were significant factors that influenced microbial community structure. DO, ${\text{NH}}_{4}^{+}$-N, ${\text{NO}}_{2}^{-}$-N, ${\text{NO}}_{3}^{-}$-N, ${\text{PO}}_{4}^{3-}$-P, and N/P ratio were not significantly correlated with the microbial community structure.

Analyses based on ABT models were used to interpret the relative importance of environmental factors and spatial isolation relative to the diversity of microbial communities in shrimp cultural enclosure ecosystems. The results indicated that temperature was the major factor affecting both phylogenetic diversity and phylotypes, accounting for over 30% of the relative influence (Figure 4). In comparison, spatial isolation, represented by gradients of latitude (accounting for about 5% of the relative influence) and longitude (accounting for <1% of the relative influence), contribution little, thus indicating that geographical distance had no obvious impact on the phylogenetic diversity and phylotypes of water microbial communities in shrimp cultural enclosure ecosystems. A CCA was further performed to examine the relationships between microbial community structures and environmental factors (Figure 5). Of all of the environmental factors examined, salinity (*r*^2^ = 0.971, *P* = 0.001), TP (*r*^2^ = 0.473, *P* = 0.004), TN (*r*^2^ = 0.428, *P* = 0.005), temperature (*r*^2^ = 0.423, *P* = 0.007), and pH (*r*^2^ = 0.380, *P* = 0.016) were significantly correlated with microbial community structure. Other environmental factors were not obviously correlated with microbial community structure (*P* > 0.05 in all cases). The results indicated that salinity was the most important environmental factor (i.e., the longest arrow) shaping the structure of microbial communities.

We also performed Welch's *t*-test to identify all OTUs that were differentially distributed between high salinity and low salinity samples. The high salinity samples were associated with the overgrowth of Chlorobi OTU5, Candidatus *Aquiluna* OTU19, Microbacteriaceae OTU21, Actinomycetales OTU28, *Marivita* OTU33, *Synechococcus* OTU39, Gammaproteobacteria OTU41, Sinobacteraceae OTU56, Nitriliruptoraceae OTU66, *Synechococcus* OTU71, Cryomorphaceae OTU96, Rhodobacteraceae OTU1412, and Chthoniobacteraceae OTU2555 (Figure S2). Furthermore, high salinity samples were associated with the depression of Actinomycetales OTU32, *Rubrivivax* OTU50, *Rhodobacter* OTU58, Pirellulaceae OTU280, and Chthoniobacteraceae OTU12327 (Figure S2). At the genus level, the high salinity samples were associated with the overgrowth of *Synechococcus* and Candidatus *Aquiluna*, and the samples were associated with the depression of *Rhodobacter, Pseudomonas, Rubrivivax*, and *Flavobacterium* (Figure S3).

### Potential functional consequences

**Figure 6:**
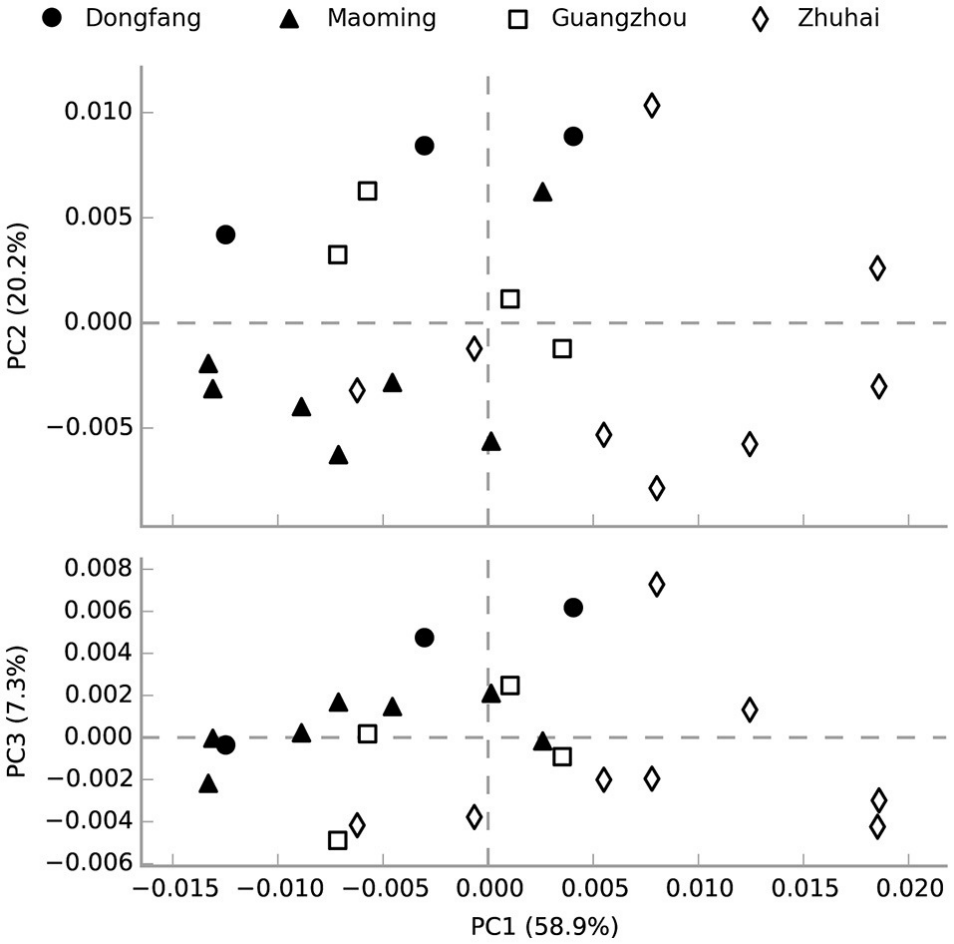
Principal component analysis (PCA) of the microbial community function content similarity based on metagenomic functional (Level 3) predictions. Black-filled symbols represent high salinity samples, and open symbols represent low salinity samples. Samples were divided into two groups based on salinity but not spatial distance.

The functional profiling of water microbial communities from all samples was predicted from 16S rRNA gene amplicon data using PICRUSt. Welch's *t*-test results (Figure S4) indicated that several predicted pathways were significantly enriched (95% confidence intervals, *P* < 0.05) in high salinity microbial communities, especially those genes associated with genetic information processing (e.g., aminoacyl-tRNA biosynthesis, DNA repair and recombination proteins, ribosome and homologous recombination) and metabolism (e.g., glycine, serine and threonine metabolism, terpenoid backbone biosynthesis, etc.). Genes associated with cellular processes (bacterial motility proteins, bacterial chemotaxis, and flagellar assembly), environmental information processing (e.g., secretion system, two-component system, and bacterial secretion system), genetic information processing (e.g., transcription factors), and metabolism (e.g., protein kinases, benzoate degradation, and butanoate metabolism) were significantly (95% confidence intervals, *P* < 0.05) more abundant in low than high salinity microbial communities. Furthermore, a PCA analysis was conducted to analyze the functional content similarity of all samples (i.e., data explained 86.4% of the variation), and the results indicated that high salinity samples were clearly separated from low salinity samples (Figure 6). Therefore, the results indicated that salinity was the most significant environmental factor that influenced the function of water microbial communities in shrimp cultural enclosure ecosystems.

## Discussion

The aim of the study was to investigate microbial community patterns and understand which environmental factors shape the structure and function of water microbial communities in shrimp cultural enclosure ecosystems. We found that environmental factors (e.g., salinity, temperature, pH, TP, and TN) largely shaped water microbial community structure, and that salinity was a major factor that shape water microbial communities in terms of structure and function in shrimp cultural enclosure ecosystems. These results generally support our hypotheses.

Our first hypothesis posited that salinity or temperature might be the major environmental factor shaping water microbial community structure. The large number of samples surveyed and the sequencing depth provided by high-throughput sequencing generated a large amount of microbial 16S rRNA gene sequencing data from enclosed aquaculture ponds water, which greatly exceeded the number of sequences reported in previous studies (Xiong et al., 2014; Dabade et al., 2016; Fan et al., 2016), thus expanding our knowledge of broad trends in water microbial community patterns in shrimp cultural enclosure ecosystems. Within the examined samples, microbial community diversity was higher than that found in previous studies of water microbial communities in different ecosystems, including tilapia ponds (Fan et al., 2016) and in estuary reservoir (Sun et al., 2014). The difference in numbers of OTUs in the waters of enclosed aquaculture ecosystems may be the results of different regions and the use of different methods. In our study, Proteobacteria was the most abundant phylum, and this was consistent with previous studies of tilapia ponds (Fan et al., 2016), Chinese grass carp ponds (Zhou et al., 2014), and shrimp ponds (Xiong et al., 2014), which exhibited an average proportion of more than 30%. The four other most abundant phyla were Bacteroidetes, Cyanobacteria, Planctomycetes, and Actinobacteria. Bacteroidetes, Planctomycetes, and Actinobacteria were always detected as the dominant phyla in shrimp cultural enclosure ecosystems (Xiong et al., 2014; Fan et al., 2016), but the distribution patterns of some phyla differed from previous observations. For example, our results showed that Cyanobacteria was a dominant phyla, but this was inconsistent with the results of shrimp ponds (Xiong et al., 2014; Dabade et al., 2016). Furthermore, in the present study, the relative abundance of Chlorobi made it a dominant phylum, but its relative abundance in other studies was low (Sakami et al., 2008; Xiong et al., 2014).

**Figure 7:**
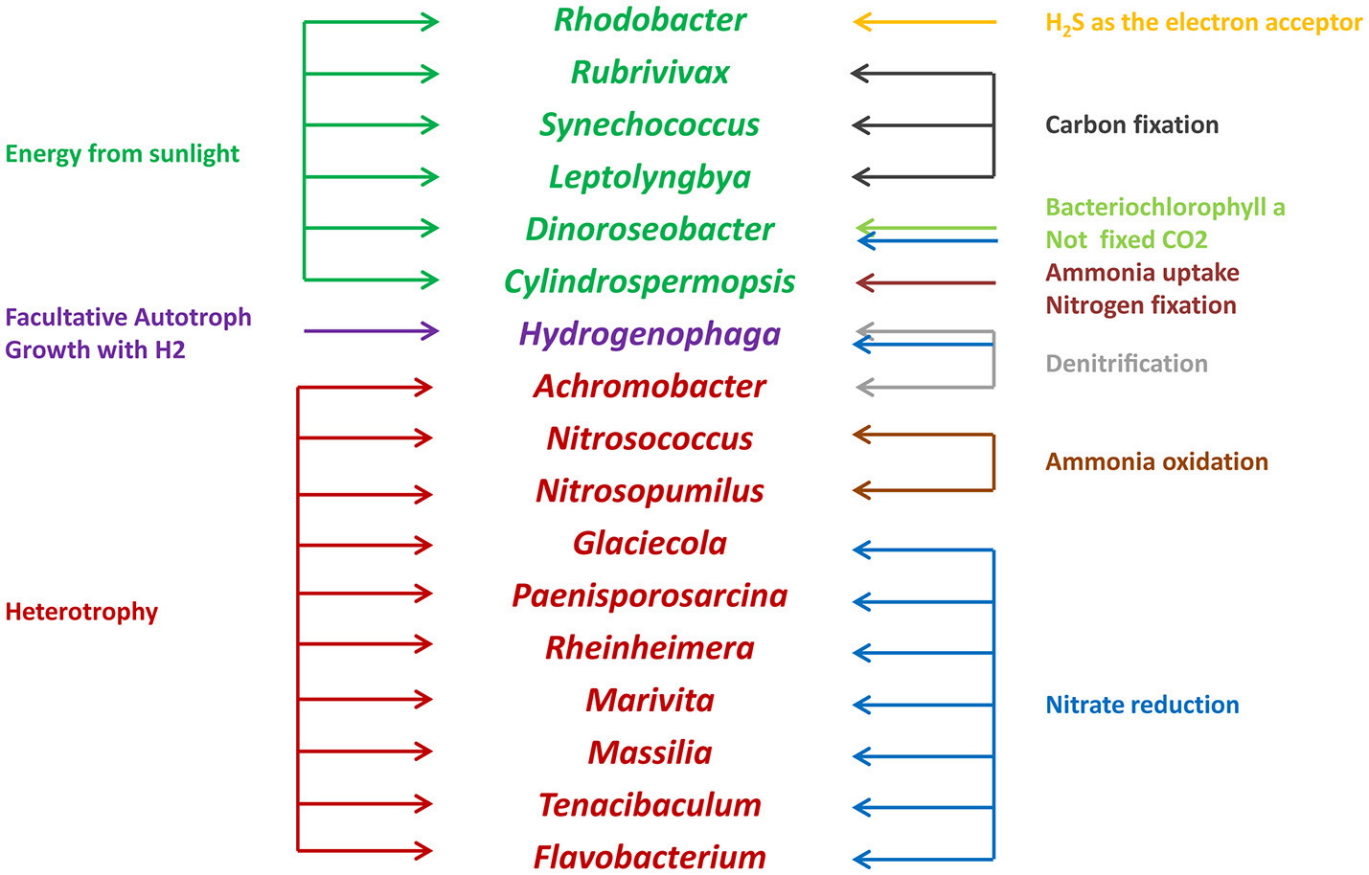
Trophic categories and classifiable functions of water microbial communities at the genus level in shrimp cultural enclosure ecosystems.

Microbes are an important component in shrimp cultural enclosure ecosystems (Moriarty, 1997). In our study, we assigned the trophic categories and functions of classifiable microbes at the genus level (Figure 7). First, some oxygenic photosynthetic microbes (capable of acquiring energy form sunlight) were detected, including *Synechococcus* (Murphy and Haugen, 1985), *Rhodobacter* (Chang et al., 1991), and *Leptolyngbya* (van der Grinten et al., 2005). Furthermore, we discovered anoxygenic phototrophic bacteria that harvest light energy such as *Dinoroseobacter* (Biebl et al., 2005). Unlike photosynthetic microbes, this organismal division is unable to fix CO_2_ using bacteriochlorophyll α and associated photosynthetic reaction centers (Moran, 2015). Other microbes are able to obtain energy from the oxidation of inorganic compounds including *Rubrivivax* (Maness et al., 2002) and *Hydrogenophaga* (Kämpfer et al., 2005), which depend on carbon monoxide oxidation pathways and the oxidation of thiosulfate to sulfide, respectively. In addition, many microbes involved in the nitrogen cycle were detected in this study. Nitrogen controls primary production of widespread N_2_-fixing Cyanobacteria such as *Cylindrospermopsis* (Padisák, 1997). *Nitrosococcus* and *Nitrosopumilus* perform the oxidation of ammonia to nitrite (Feng et al., 2016), so they are referred to as ammonia-oxidizing bacteria. *Glaciecola, Paenisporosarcina, Rheinheimera, Marivita, Massilia*, and *Flavobacterium* are associated with function of nitrate reduction (Bernardet et al., 1996; Baik et al., 2006; Zhang et al., 2006; Yoon et al., 2007, 2013; Reddy et al., 2013). As denitrifying bacteria, *Achromobacter* are involved in the conventional anaerobic denitrification process (Gui et al., 2017), some microbes such as *Hydrogenophaga* perform nitrification and denitrification at the same time (Kämpfer et al., 2005). Moreover, some pathogens such as *Pseudomona, Vibrio*, and *Flavobacterium*, which may be related to the diseases of cultured animals (Dang and Lovell, 2016), were detected in our study. We also found that some functional groups were exhibited significantly different abundances in high and low salinity waters. Compared to low salinity water, the relative abundance of *Synechococcus* was overrepresented in high salinity, and the relative abundances of *Rhodobacter* and *Flavobacterium* were reduced. These results suggested that microbes play key roles with respect to the productivity, nutrient cycling and water quality of shrimp cultural enclosure ecosystems.

Salinity and temperature are key environmental factors that shape microbial community structure in different environments (Lozupone and Knight, 2007; Sunagawa et al., 2015). Nevertheless, previous studies have reported that salinity, rather than temperature, explained a significant portion of global microbial community distribution patterns (Lozupone and Knight, 2007; Auguet et al., 2010). In our study, the results also showed that salinity was the primary environmental factor that shaped water microbial community divergence in shrimp cultural enclosure ecosystems, but temperature was not a primary factor. Moreover, we found that the differences in microbial community structure between high and low salinity samples were explained by the changes in the relative abundances of some OTUs (e.g., OTU5, OTU19, OTU21, OTU39, and OTU71) thus indicating that salinity may affect the distribution of these microbes. Further study of the relationship between high/low salinity and microbial communities is going on. However, it should be noted that the influence of salinity and temperature on microbial community structure does not completely negate the influence of other environmental factors. Particularly, TP and TN concentrations were significantly correlated with the microbial community structure examined in this study, and this result was not observed studies of in other environments (Xiong et al., 2012; Kuang et al., 2013; Liu et al., 2014). Furthermore, pH was the primary environmental factor determining the microbial community structure in various environment types (Shen et al., 2013; Liu et al., 2015). Our results indicated that pH was important, but it was not a primary factor in the enclosed aquaculture ecosystems. In addition, some other environmental factors such as dissolved organic matter (DOM) and particulate organic matter (POM) are important environmental factors that affect microbial community composition and structure in marine waters (Dang and Lovell, 2016). Our study focused on the relationship between various environmental factors (e.g., salinity, temperature, TN, TP, pH, DO, ${\text{NH}}_{4}^{+}$-N, ${\text{NO}}_{2}^{-}$-N, ${\text{NO}}_{3}^{-}$-N, ${\text{PO}}_{4}^{3-}$-P, and geographical distance) and the microbial community structure in shrimp cultural enclosure ecosystems, and the further ongoing work will examine the relationship between additional environmental factors and microbial community structure. The microbial community structure may be affected by antibiotic applications in shrimp cultural enclosure ecosystems (Xi et al., 2015). In the present study, the samples were collected from 22 shrimp cultural ponds, which were in strict accordance with national aquaculture regulations, which antibiotic applications during the shrimp culture period. Moreover, we are currently conducting other studies that focus on the effects of antibiotics in the surrounding waters and intestinal microbial community of shrimp and the presence of antibiotic resistance genes (ARGs) in surrounding waters and intestines (unpublished data). Furthermore, water environmental factors (e.g. salinity) and shifts in microbial community structure may be related to shrimp production, so further studies will focus on environmental factors and corresponding shifts in microbial community structures that affect shrimp production.

Our second hypothesis was that salinity would also affect the function of microbial communities. Salinity and temperature differences can also affect the function of microbial communities (Székely et al., 2013; Sunagawa et al., 2015). A previous analysis of functional trait compositions in ocean environments suggested that temperature had stronger effects than salinity (Sunagawa et al., 2015). Whereas, our results revealed that salinity rather than temperature explained a significant portion of microbial community function in shrimp cultural enclosure ecosystems. This difference may be caused by the wide range of salinity (0.42–32.71‰) but the narrow range of temperature (from 23.84 to 32.40°C) detected in shrimp cultural enclosure ecosystems. Regarding microbes, the main factors that determine whether certain types of microbial communities survive at high salt concentrations are the amount of energy generated during its metabolism and osmotic adaptation (Oren, 2011). However, salinity can affect microbial community production by enhancing nutrient availability (Pinckney et al., 1995). Accordingly, in our study, genes associated with energy metabolism (e.g., glycine, serine and threonine metabolism, fructose and mannose metabolism, photosynthesis, purine metabolism, etc.) were significantly more abundant in high salinity than in low salinity conditions, thus revealing that the function of the microbial community significantly contributed to responses to salinity changes in shrimp cultural enclosure ecosystems. The differences between these functions in waters of shrimp cultural enclosure ecosystems with different salinity conditions will be studied in the future.

## Conclusion

This study represents an attempt to investigate water microbial community patterns in shrimp cultural enclosure ecosystems. Our results clearly showed that the major environmental determinant of microbial community variation was salinity rather than temperature, TP, TN, and pH. We also showed that salinity was a major contributor to the function of the water microbial community. The results provide baseline information for predicting the structure and function of microbial community responses to environmental changes in shrimp cultural enclosure ecosystems.

## Author contributions

DH, SZ, JL, DW, and XD: performed the experiments; DH and SZ: analyzed the data. DH, ZhijianH, and ZhiliH: wrote the paper; DH, ZhijianH, SW, and JH: contributed the conception of the work. DH and ZhijianH: critically revised important content in the manuscript. JH was primarily responsible for the final content.

### Conflict of interest statement

The authors declare that the research was conducted in the absence of any commercial or financial relationships that could be construed as a potential conflict of interest.

## Supplementary material

Figure S1 Relative abundances (%) of genera from all samples based on 16S rRNA gene amplicon sequencing data.

Figure S2 Extended error bar plot identifying significant differences between mean proportions of OTUs in high salinity (black) and low salinity (white) samples (95% confidence intervals). Corrected *p-*values are shown on the right.

Figure S3 Extended error bar plot identifying significant differences between mean proportions of genera in high salinity (black) and low salinity (white) samples (95% confidence intervals). Corrected *p-*values are shown on the right.

Figure S4 Extended error bar plot identifying significant differences between mean proportions of functional genes in high salinity (black) and low salinity (white) samples (95% confidence intervals). Corrected *p-*values are shown on the right.

Table S1 Sampling sites characteristics. Samples A, B, C, D, E, F, and G were collected from Maoming City, samples H, I, J, K, L, M, N, and O were collected from Zhuhai City, samples P, Q, R, and S were collected from Guangzhou City, samples T, U, and V were collected from Dongfang City.

Table S2 The environmental factors of all water samples collected in shrimp cultural enclosure ecosystems.

Table S3 Summary of high-throughput sequencing read analysis, microbial community diversity richness (OTUs, 97%), sample coverage (Good's coverage), diversity index (Shannon), and estimated OTU richness (Chao1) for prokaryotic community diversity analyses of 66 water samples from 22 culture ponds.
