# Supplementary figures and images for: Environmental Factors Shape Water Microbial Community Structure and Function in Shrimp Cultural Enclosure Ecosystems
Source: Front Microbiol. 2017 Nov 29;8:2359. doi: 10.3389/fmicb.2017.02359 (PMC5712584; doi:10.3389/fmicb.2017.02359)

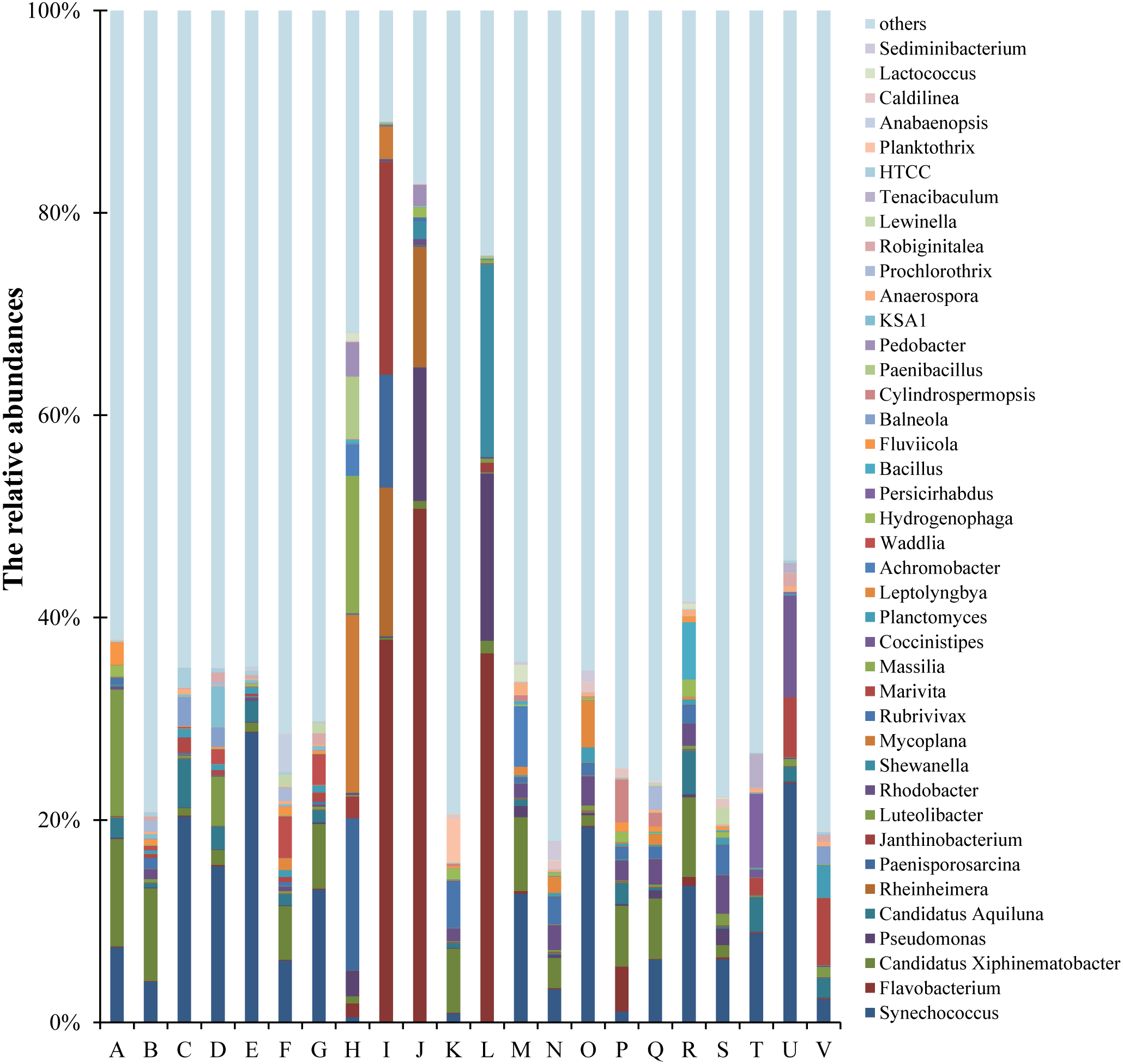

Supplement: Figure S1 — Relative abundances (%) of genera from all samples based on 16S rRNA gene amplicon sequencing data. [file Image1.TIF]

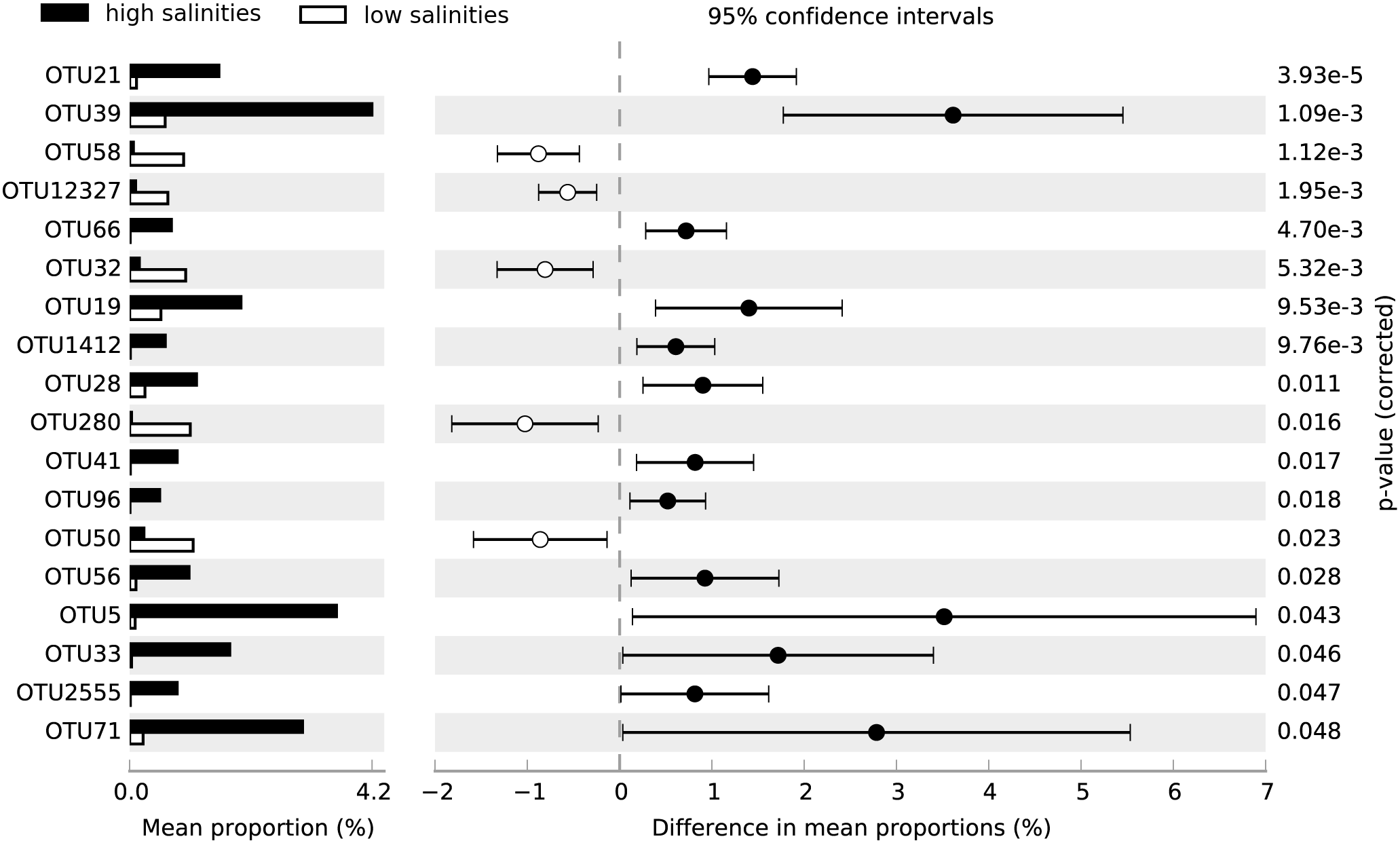

Supplement: Figure S2 — Extended error bar plot identifying significant differences between mean proportions of OTUs in high salinity (black) and low salinity (white) samples (95% confidence intervals). Corrected p-values are shown on the right. [file Image2.TIF]

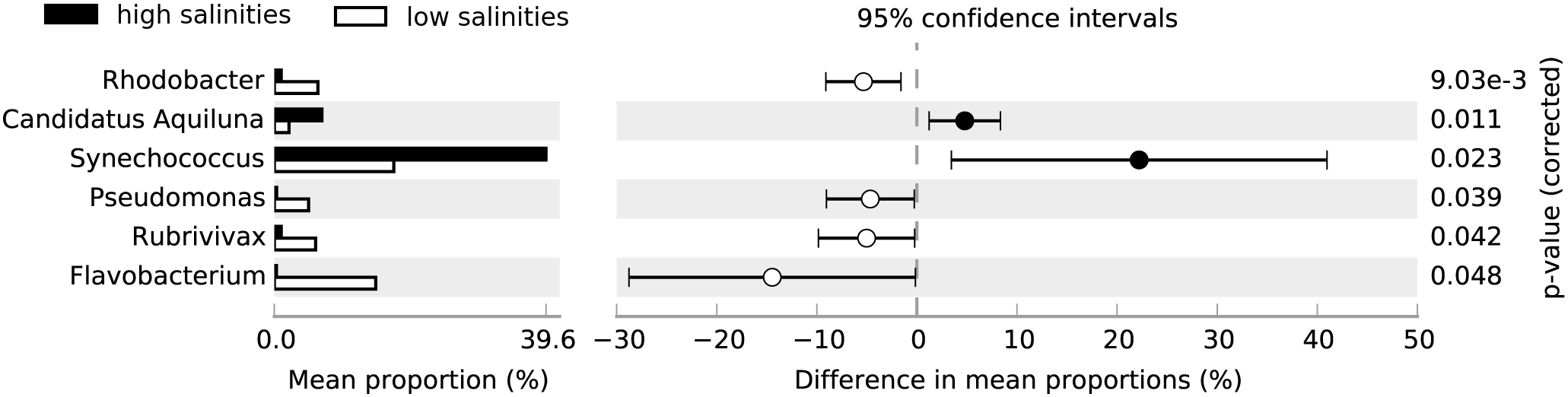

Supplement: Figure S3 — Extended error bar plot identifying significant differences between mean proportions of genera in high salinity (black) and low salinity (white) samples (95% confidence intervals). Corrected p-values are shown on the right. [file Image3.TIF]

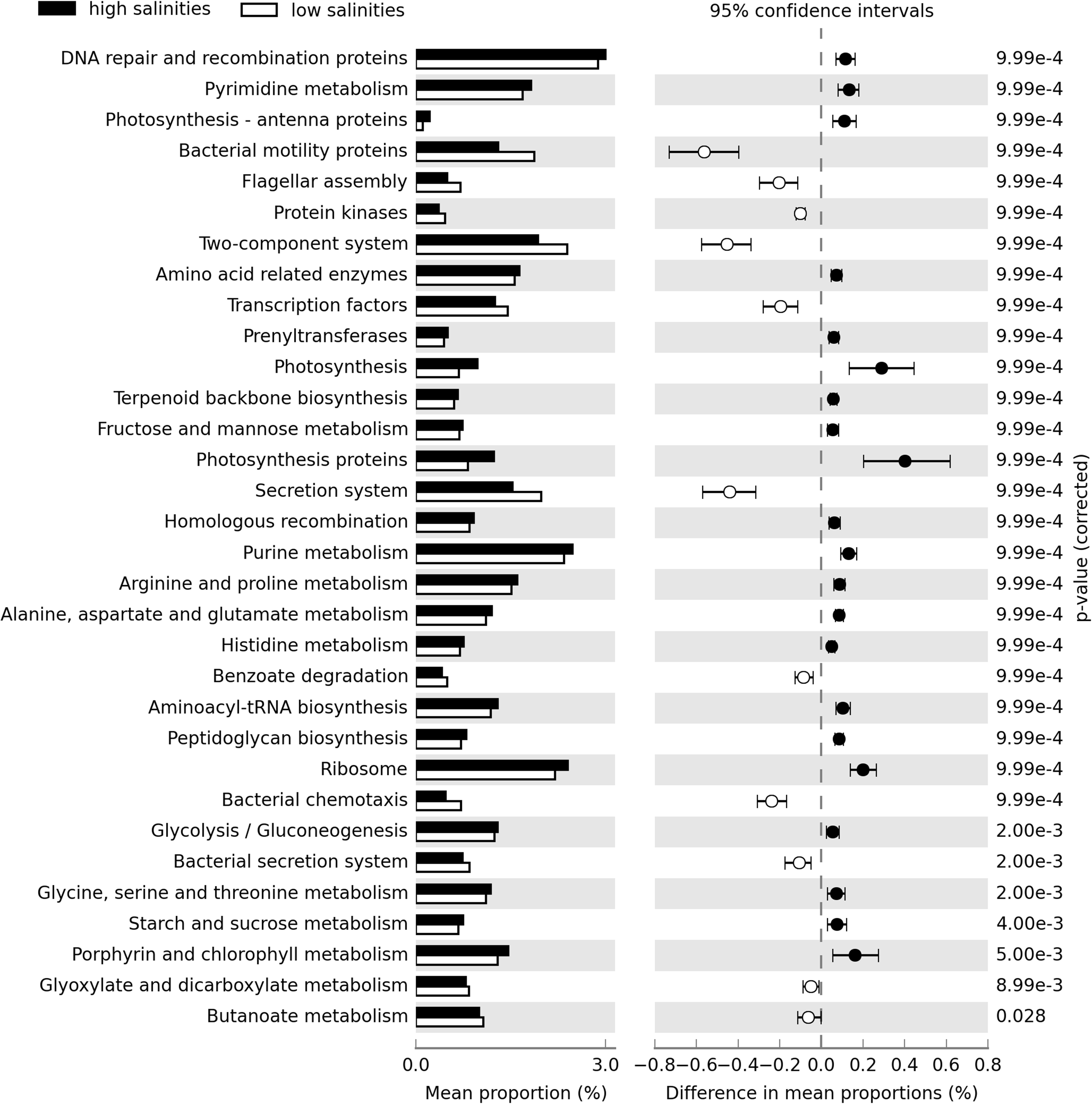

Supplement: Figure S4 — Extended error bar plot identifying significant differences between mean proportions of functional genes in high salinity (black) and low salinity (white) samples (95% confidence intervals). Corrected p-values are shown on the right. [file Image4.TIF]
