# Supplementary material for: Environmental Factors Shape Water Microbial Community Structure and Function in Shrimp Cultural Enclosure Ecosystems
Source: Front Microbiol. 2017 Nov 29;8:2359. doi: 10.3389/fmicb.2017.02359 (PMC5712584; doi:10.3389/fmicb.2017.02359)
Supplement: Table S1 — Sampling sites characteristics. Samples A, B, C, D, E, F, and G were collected from Maoming City, samples H, I, J, K, L, M, N, and O were collected from Zhuhai City, samples P, Q, R, and S were collected from Guangzhou City, samples T, U, and V were collected from Dongfang City. [file Table1.DOCX]

| **Sample Number** | **Province** | **City** | **Date (mm/dd/yyyy)** | **Latitude (◦N)** | **Longitude (◦E)** | **Area (m^2^)** |
| --- | --- | --- | --- | --- | --- | --- |
| **A** | **Guangdong** | Maoming | 08/03/2016 | N21.517801 | E111.389937 | 3,300 |
| **B** |  |  | 08/21/2016 | N21.517801 | E111.389937 | 3,300 |
| **C** |  |  | 08/21/2016 | N21.545445 | E111.399402 | 3,300 |
| **D** |  |  | 08/21/2016 | N21.545445 | E111.399402 | 3,300 |
| **E** |  |  | 08/23/2016 | N21.545445 | E111.399402 | 3,300 |
| **F** |  |  | 08/23/2016 | N21.517801 | E111.389937 | 3,300 |
| **G** |  |  | 08/23/2016 | N21.517801 | E111.389937 | 3,300 |
| **H** |  | Zhuhai | 08/06/2016 | N22.354679 | E113.598694 | 2,700 |
| **I** |  |  | 08/06/2016 | N22.354679 | E113.598694 | 2,700 |
| **J** |  |  | 09/07/2016 | N22.135308 | E113.229959 | 2,700 |
| **K** |  |  | 09/07/2016 | N22.135308 | E113.229959 | 2,700 |
| **L** |  |  | 09/07/2016 | N22.135308 | E113.229959 | 2,700 |
| **M** |  |  | 09/07/2016 | N22.044768 | E113.28565 | 1,300 |
| **N** |  |  | 09/07/2016 | N22.044768 | E113.28565 | 1,300 |
| **O** |  |  | 09/07/2016 | N22.044768 | E113.28565 | 1,300 |
| **P** |  | Guangzhou | 10/12/2016 | N22.963846 | E113.531164 | 2,700 |
| **Q** |  |  | 10/12/2016 | N22.963846 | E113.531164 | 2,700 |
| **R** |  |  | 10/12/2016 | N22.963846 | E113.531164 | 2,700 |
| **S** |  |  | 10/12/2016 | N22.963846 | E113.531164 | 2,700 |
| **T** | **Hainan** | Dongfang | 08/07/2016 | N18.860713 | E108.656068 | 1,300 |
| **U** |  |  | 08/07/2016 | N18.854978 | E108.650731 | 1,300 |
| **V** |  |  | 08/07/2016 | N18.854978 | E108.650731 | 1,300 |
