# Supplementary material for: Environmental Factors Shape Water Microbial Community Structure and Function in Shrimp Cultural Enclosure Ecosystems
Source: Front Microbiol. 2017 Nov 29;8:2359. doi: 10.3389/fmicb.2017.02359 (PMC5712584; doi:10.3389/fmicb.2017.02359)
Supplement: Table S2 — The environmental factors of all water samples collected in shrimp cultural enclosure ecosystems. [file Table2.DOCX]

| **Sample number** | **TN (mg^.^L^-1^)** | **TP (mg^.^L^-1^)** | **N/P** | **NH_4_^+^-N (mg^.^L^-1^)** | **NO_2_^-^-N (mg^.^L^-1^)** | **NO_3_^-^-N (mg^.^L^-1^)** | **PO_4_^3-^-P (mg^.^L^-1^)** | **pH** | **Salinity**  **(‰)** | **DO (mg^.^L^-1^)** | **T (**ºC**)** |
| --- | --- | --- | --- | --- | --- | --- | --- | --- | --- | --- | --- |
| **A** | 1.55 | 0.65 | 2.38 | 1.23 | 0.02 | 0.09 | 0.34 | 8.28 | 4.47 | 5.22 | 29.20 |
| **B** | 1.02 | 1.02 | 1.00 | 0.01 | 0.02 | 0.08 | 0.23 | 8.35 | 7.56 | 5.04 | 29.60 |
| **C** | 1.91 | 0.75 | 2.55 | 0.19 | 0.06 | 0.06 | 0.09 | 9.05 | 11.76 | 6.37 | 27.70 |
| **D** | 1.02 | 0.62 | 1.65 | 0.10 | 0.03 | 0.03 | 0.02 | 9.21 | 13.35 | 7.79 | 29.10 |
| **E** | 2.58 | 0.78 | 3.31 | 1.69 | 0.04 | 0.13 | 0.06 | 9.07 | 10.76 | 7.30 | 28.10 |
| **F** | 2.41 | 0.68 | 3.54 | 0.11 | 0.09 | 0.02 | 0.02 | 9.81 | 6.89 | 19.04 | 32.40 |
| **G** | 2.36 | 1.32 | 1.79 | 1.48 | 0.10 | 0.06 | 0.29 | 9.21 | 7.16 | 11.09 | 31.60 |
| **H** | 1.18 | 0.22 | 5.36 | 0.06 | 0.13 | 0.20 | 0.13 | 8.42 | 1.04 | 5.21 | 31.28 |
| **I** | 0.75 | 0.22 | 3.41 | 0.04 | 0.12 | 0.10 | 0.11 | 8.80 | 1.03 | 6.79 | 31.48 |
| **J** | 3.81 | 0.19 | 20.04 | 2.60 | 0.02 | 0.14 | 0.20 | 7.61 | 1.64 | 3.45 | 29.89 |
| **K** | 4.55 | 0.01 | 379.42 | 0.18 | 0.01 | 0.06 | 0.07 | 7.82 | 1.73 | 4.66 | 29.83 |
| **L** | 2.83 | 0.01 | 217.54 | 0.06 | 0.01 | 0.06 | 0.05 | 7.31 | 0.42 | 3.60 | 29.89 |
| **M** | 5.14 | 0.12 | 42.84 | 2.49 | 0.20 | 0.64 | 0.17 | 8.84 | 1.12 | 5.70 | 29.41 |
| **N** | 5.15 | 0.08 | 64.35 | 2.14 | 0.01 | 0.03 | 0.27 | 8.36 | 0.56 | 5.57 | 28.88 |
| **O** | 5.16 | 0.71 | 7.27 | 2.50 | 0.37 | 0.87 | 0.12 | 8.43 | 0.58 | 6.07 | 28.79 |
| **P** | 5.14 | 0.69 | 7.45 | 0.71 | 0.02 | 2.08 | 0.12 | 7.85 | 1.99 | 4.55 | 23.84 |
| **Q** | 5.15 | 0.30 | 17.18 | 0.44 | 0.01 | 0.06 | 0.05 | 8.43 | 1.57 | 5.02 | 24.16 |
| **R** | 5.11 | 0.22 | 23.22 | 3.24 | 0.13 | 0.40 | 0.56 | 8.45 | 2.27 | 5.34 | 24.02 |
| **S** | 5.15 | 0.18 | 28.63 | 2.51 | 0.07 | 0.23 | 0.58 | 8.29 | 0.63 | 5.33 | 23.99 |
| **T** | 0.60 | 0.16 | 3.75 | 0.08 | 0.07 | 0.01 | 0.07 | 8.42 | 29.23 | 5.45 | 32.33 |
| **U** | 1.87 | 1.05 | 1.78 | 0.19 | 0.34 | 0.51 | 0.18 | 8.10 | 29.51 | 6.07 | 32.16 |
| **V** | 1.89 | 0.82 | 2.30 | 0.17 | 0.24 | 0.22 | 0.22 | 8.23 | 32.71 | 7.16 | 31.74 |
