# Supplementary material for: Environmental Factors Shape Water Microbial Community Structure and Function in Shrimp Cultural Enclosure Ecosystems
Source: Front Microbiol. 2017 Nov 29;8:2359. doi: 10.3389/fmicb.2017.02359 (PMC5712584; doi:10.3389/fmicb.2017.02359)
Supplement: Table S3 — Summary of high-throughput sequencing read analysis, microbial community diversity richness (OTUs, 97%), sample coverage (Good's coverage), diversity index (Shannon), and estimated OTU richness (Chao1) for prokaryotic community diversity analyses of 66 water samples from 22 culture ponds. [file Table3.DOCX]

|  |  | | | **Diversity index** | | |  |  | | | **Diversity index** | | |
| --- | --- | --- | --- | --- | --- | --- | --- | --- | --- | --- | --- | --- | --- |
|  | **Sequences** | **OTUs** | **Phylum** | **GC** | **Shannon** | **Chao1** |  | **Sequences** | **OTUs** | **Phylum** | **GC** | **Shannon** | **Chao1** |
| **A1** | 32,484 | 1,146 | 28 | 0.97 | 5.85 | 1,696 | **L1** | 44,997 | 998 | 25 | 0.97 | 4.93 | 1,112 |
| **A2** | 34,838 | 1,132 | 30 | 0.95 | 5.90 | 1,632 | **L2** | 43,521 | 913 | 25 | 0.97 | 4.51 | 1,170 |
| **A3** | 30,395 | 1,139 | 31 | 0.97 | 5.80 | 1,687 | **L3** | 41,074 | 986 | 22 | 0.97 | 5.41 | 1,082 |
| **B1** | 32,376 | 1,438 | 34 | 0.97 | 6.77 | 1,909 | **M1** | 38,166 | 1,604 | 27 | 0.95 | 7.21 | 1,963 |
| **B2** | 29,193 | 1,275 | 32 | 0.97 | 6.63 | 1,962 | **M2** | 41,002 | 1,498 | 30 | 0.97 | 6.86 | 1,702 |
| **B3** | 31,400 | 1,438 | 31 | 0.97 | 6.78 | 2,019 | **M3** | 44,341 | 1,531 | 36 | 0.97 | 6.62 | 1,595 |
| **C1** | 38,793 | 1,339 | 31 | 0.97 | 6.40 | 1,638 | **N1** | 32,418 | 1,544 | 27 | 0.97 | 7.22 | 1,883 |
| **C2** | 33,265 | 1,205 | 30 | 0.97 | 6.61 | 1,552 | **N2** | 32,146 | 1,518 | 27 | 0.97 | 7.18 | 2,060 |
| **C3** | 39,385 | 1,315 | 30 | 0.97 | 6.49 | 1,807 | **N3** | 27,160 | 1,532 | 26 | 0.97 | 7.25 | 2,221 |
| **D1** | 37,449 | 1,325 | 32 | 0.97 | 6.77 | 1,576 | **O1** | 45,360 | 1,835 | 31 | 0.97 | 7.28 | 2,087 |
| **D2** | 37,114 | 1,255 | 32 | 0.95 | 6.94 | 1,574 | **O2** | 40,889 | 1,733 | 29 | 0.97 | 7.03 | 2,040 |
| **D3** | 35,752 | 1,282 | 30 | 0.97 | 6.84 | 1,805 | **O3** | 41,267 | 1,695 | 26 | 0.95 | 6.88 | 1,954 |
| **E1** | 35,498 | 1,573 | 39 | 0.97 | 6.40 | 2,228 | **P1** | 32,227 | 1,252 | 30 | 0.97 | 6.83 | 1,704 |
| **E2** | 31,344 | 1,436 | 41 | 0.97 | 6.38 | 2,013 | **P2** | 33,929 | 1,242 | 27 | 0.97 | 6.76 | 1,612 |
| **E3** | 32,348 | 1,499 | 39 | 0.97 | 6.53 | 2,125 | **P3** | 36,047 | 1,259 | 35 | 0.97 | 6.78 | 1,665 |
| **F1** | 26,346 | 1,361 | 33 | 0.97 | 7.27 | 2,074 | **Q1** | 38,400 | 1,412 | 34 | 0.97 | 6.66 | 1,544 |
| **F2** | 25,520 | 1,331 | 34 | 0.97 | 7.22 | 2,070 | **Q2** | 39,883 | 1,304 | 32 | 0.97 | 6.41 | 1,539 |
| **F3** | 28,910 | 1,422 | 34 | 0.97 | 7.26 | 1,943 | **Q3** | 38,062 | 1,226 | 34 | 0.97 | 6.55 | 1,415 |
| **G1** | 28,321 | 1,332 | 34 | 0.97 | 6.75 | 1,943 | **R1** | 36,864 | 1,304 | 32 | 0.97 | 6.99 | 1,561 |
| **G2** | 28,226 | 1,327 | 32 | 0.97 | 6.77 | 1,923 | **R2** | 35,746 | 1,331 | 35 | 0.95 | 7.07 | 1,591 |
| **G3** | 28,045 | 1,315 | 31 | 0.95 | 6.69 | 1,920 | **R3** | 34,370 | 1,221 | 30 | 0.97 | 6.23 | 1,607 |
| **H1** | 41,004 | 1,020 | 27 | 0.97 | 5.34 | 1,153 | **S1** | 31,192 | 1,337 | 35 | 0.97 | 7.94 | 1,617 |
| **H2** | 45,804 | 788 | 22 | 0.97 | 4.20 | 903 | **S2** | 30,150 | 1,447 | 38 | 0.97 | 7.88 | 1,832 |
| **H3** | 45,784 | 814 | 21 | 0.97 | 4.13 | 1,071 | **S3** | 24,548 | 1,247 | 36 | 0.97 | 7.81 | 1,687 |
| **I1** | 46,229 | 659 | 20 | 0.97 | 3.69 | 702 | **T1** | 35,740 | 1,259 | 33 | 0.97 | 6.14 | 1,749 |
| **I2** | 52,304 | 741 | 22 | 0.97 | 3.48 | 748 | **T2** | 32,748 | 1,187 | 31 | 0.97 | 6.26 | 1,724 |
| **I3** | 46,295 | 700 | 22 | 0.97 | 3.54 | 1,026 | **T3** | 34,835 | 1,432 | 31 | 0.97 | 6.59 | 1,843 |
| **J1** | 50,917 | 948 | 25 | 0.97 | 3.89 | 1,192 | **U1** | 37,209 | 1,512 | 30 | 0.95 | 6.70 | 1,879 |
| **J2** | 42,071 | 899 | 25 | 0.95 | 4.08 | 1,281 | **U2** | 34,542 | 1,440 | 31 | 0.97 | 6.58 | 1,723 |
| **J3** | 54,163 | 1,020 | 29 | 0.97 | 4.10 | 1,314 | **U3** | 34,170 | 1,323 | 29 | 0.97 | 6.33 | 1,781 |
| **K1** | 33,111 | 1,705 | 46 | 0.97 | 7.19 | 2,401 | **V1** | 25,689 | 1,462 | 28 | 0.97 | 7.45 | 1,888 |
| **K2** | 27,187 | 1,761 | 45 | 0.97 | 7.35 | 2,593 | **V2** | 22,954 | 1,352 | 32 | 0.97 | 7.50 | 1,854 |
| **K3** | 28,780 | 1,804 | 45 | 0.97 | 7.47 | 2,731 | **V3** | 17,745 | 1,215 | 28 | 0.97 | 7.51 | 1,846 |
